# What place does elite sport have for women? A scoping review of constraints

**DOI:** 10.3389/fspor.2023.1121676

**Published:** 2023-06-14

**Authors:** Kotryna K. Fraser, Jill Kochanek

**Affiliations:** ^1^ School of Health Sciences, Faculty of Medicine and Health, The University of Sydney, Camperdown, NSW, Australia; ^2^ Department of Physical Education and Health, Springfield College, Springfield, MA, United States

**Keywords:** female athlete, female sport, gender stereotypes, equity in sport, cultural sport psychology, constraints-led approach, Newell's model

## Abstract

**Introduction:**

Despite increases in participation and raised attention for girls and women in sports, female sport is still based on male evidence that ignores gendered differences and experiences of unequal treatment and marginalization from grassroots to elite sport. This paper aimed to critically interrogate the place that women have in the male preserve of elite sport by conducting a two-part study.

**Methods:**

First, we provided a brief sociohistorical analysis of gender in sport as a means to move away from a decontextualized and universalized approach dominating in sport science literature. We then conducted a scoping review following PRISMA-ScR guidelines to synthesize existing sport science literature that implemented Newell's constraints-led approach to examine elite performance.

**Results:**

Ten studies were identified, none of which collected demographic data or centred on female athletes and the effects of sociocultural constraints on their performance. Instead, male-centred, masculine sports and physiological profiles dominated the identified studies.

**Discussion:**

We discussed these results considering critical sport research and cultural sport psychology literature to offer an integrative, interdisciplinary approach to advocate for more culturally sensitive, context-specific interpretations of gender as a sociocultural constraint. We put forth a call to action for sport science researchers, practitioners, and decision-makers to move away from implementing male evidence in female sport and attend to the unique needs of female athletes. Practical suggestions aimed to help stakeholders reimagine elite sport by celebrating these [potential] differences as strengths for promoting gender equity in sport.

## Introduction

Despite the growing popularity of female sport and its use as a medium for social change, elite female sport is largely based on male evidence (1) and perspective [e.g., male coaches, reporters, photographers, or commentators] that disregard the effects gendered discourse and dynamics have on a female athlete (2, 3). These factors hinder the experiences and development of professional female athletes and put them at higher risk of lower-extremity stress fractures and overuse injuries (4, 5), higher prevalence of developing poor mental health symptoms (6, 7) or public and invasive scrutiny of their own bodies (8). These realities necessitate rethinking the place elite sport has for women to retain and develop talent in the most effective and holistic way. We can only then push the performance limits while ensuring individual flourishing.

Therefore, the overall aim of this paper is to critically interrogate *the place that elite sport has for women in the male preserve of sport.* In doing so, we undertake an interdisciplinary approach that aims to: (a) provide a brief sociohistorical analysis of gender to shed light on the deep, historically rooted sociocultural constraints that have uniquely restricted girls and women in sport and continue to do so at an elite level; (b) conduct a scoping review of sport science literature that used Newell's (9) Model of Constraints in elite performance to better understand the extent to which sociocultural constraints have been purposefully considered when examining performance factors of the elite athletes; and, (c) offer tangible examples of how sport science scholars and practitioners might more rigorously integrate gender discourse and knowledge developed by sport sociology scholars to comprehensively address *still* existing gender inequalities at the elite level. We highlight the critical need for further investments in equity (i.e., acknowledging different backgrounds and unequal access to opportunities and resources) to achieve equality in the long term and reimage *the place that elite sport has for women and girls*.

## The interdisciplinary approach

Sport sociology scholars have critically examined gender inequalities in sport for decades (10–12). Coakley (13) explained that sport, historically, was a means to express presumed masculine attributes [e.g., aggressiveness and physicality] over perceived feminine qualities [e.g., weakness and care]. Nonetheless, similar scholarly works emphasising gendered dynamics are only recently being explored among other sport science disciplines such as performance psychology [e.g. (14)] or motor development [e.g. (15)]. These studies put forth valuable information about sex-based differences in performance and development despite failing to consider historically constructed perceptions of gender and power that uniquely advantage white cisgender [i.e., gender identity matches the sex assigned at birth] able-bodied, heterosexual men and disadvantage individuals with other identities [see (10)]. On the other hand, critical sports scholars often fail to contextualise their work and advocacy by utilising original empirical works and lack more practical, tangible solutions relevant to a modern-day environment [e.g., (16)].

There is no clear, simple, yet rigorous framework used to investigate such experiences empirically that allows easy integration into applied sport science research and coaching practice. As such, we turn to Newell's (9) Model of Constraints which explains the development of motor skills by considering gendered roles and expectations, at least to some extent. Newell's model has laid the foundation for the more complex framework used in sport science, especially in skill acquisition and motor control [e.g., Ecological Dynamics Theory (17, 18)]. Far from ideal, Newell's model offers a clear and simple framework that could be used as a reference point when assessing gendered discourse and its effects on motor performance from grassroots to elite levels. It also allows us to consider possible solutions for developing skilled performers.

### Newell's (1986) model of constraints

According to Newell (9), the three constraints impose on each individual, resulting in a pattern of movement: individual, task, and environment. Individual constraint regards the individual and considers structural [i.e., physiological aspects such as height] and functional [i.e., psychological aspects such as level of motivation] components. Task constraint considers a goal of, equipment needed, and rules related to a given task. Environmental constraints pertain to external physical forces [e.g., the surface of the playing field] and sociocultural factors [e.g., gendered stereotypes] imposed on each individual. Newell (9) noted that constraints can change over time at varying rates.

Newell's model posits that the optimal pattern of coordination—or technique—and action control emerges from the interaction of *all* constraints, though one constraint may be more salient at times [e.g., lack of strength and power in younger children as an individual-structural constraint]. An individual [e.g., a coach] can manipulate constraints to enable motor development and the desired outcome. For instance, Timmerman et al. (19) reported the benefits of such practice as children playing tennis on a proportionally smaller court and net reached similar levels of game intensity recorded in adult games and indicated more satisfaction with the game. Therefore, manipulations of individual-structural and/or task constraints will *result* in changes in technique, while fixed constraints [i.e., those not being manipulated, such as sex or environment] will *explain* the observed changes. Such manipulation either hinders or facilitates development leading to expert performance and further influencing performance at elite levels.

#### Sociocultural constraints

Newell (9) defined sociocultural constraints as broad experiences consisting of different cultural backgrounds that interact with one's environment rather than act upon developing optimal motor movements. Based on limited evidence existing at that time, Newell (9) argued that manipulating sociocultural constraints was complex and suggested that overall personal experience might be less significant compared to other constraints.

Consequently, sociocultural constraints are less often manipulated, despite our evolving understanding that children experiencing sociocultural constraints cannot reach the same proficiency of fundamental—and more complex—motor skills later in life [e.g., (20)]. Goodway et al. (21) found that developmental deficiencies in girls' ball control proficiency compared to boys begin in early childhood and worsen with age. Thus, opportunities and gendered norms constrain motor development over time *leading* to deficiencies between sexes rather than these deficiencies being natural. This implies that sociocultural constraints impact one's physical environment (e.g., access to the facilities or knowledge) and the development of optimal movement patterns needed for future skilled [or elite] performances. It also alludes to the need to better understand elite performance from the constraints-based approach integrating gendered discourse and sociocultural constraints rather than continue investigating recreational participation at an amateur or grassroots level and focusing on immediate physiological differences.

These points are in line with the recent arguments that sociocultural constraints may be an exploratory variable explaining some sex differences observed between male and female athletes [e.g., (22, 23)]. Fox et al. (22) and later Parsons, Coen and Bekker (23) reviewed the existing literature and argued that sociocultural factors and gendered environments explained the higher prevalence of anterior cruciate ligament injury among elite female athletes. The scholars noted that a player exposed to daily playground physical activity from an early age, influenced by social expectations and friendship groups, and playing traditionally masculine sports, may be better prepared for the physical demands of elite-level sport including protection from injury [i.e., typically a male athlete]. Therefore, gendered norms—a type of sociocultural constraint—influence environmental constraints (e.g., the opportunity to play), lead to performance differences over a prolonged time, and are often further masked by individual structural differences taken at face value.

A significant underlying reason for overlooking sociocultural constraints and their effects on initial motor development and later developed skilled performance until recently is due to gendered societal norms, stereotypes, and expectations. Messner (10) critiqued popular beliefs which discount the relevance of historically and socially primed gender roles and dynamics. Applied to sport, such beliefs overlook the gendered practices and often reduce gender differences to biological explanations of performance disparities [i.e., naturalising the “muscle gap” (24) p. 197]. This is evident in the perceived gender-appropriateness of sport as males are encouraged to participate in masculine sports [i.e., sports associated with strength] while females are more supported to participate in feminine sports [i.e., sports associated with aesthetics (25)]. Such categorisation of sport is based on long-standing gendered roles that uphold stereotypes and restrict child's development due to choices made based on their sex.

Mainstream sports science is not immune to this gendered bias as only recently more research on a female athlete has emerged alongside considerations given to their gendered environments as potential covariables [see (3, 22, 26, 27)]. Indeed, Larsson's (28) review highlighted that despite researchers' advocacy for women “to do sport *on their own term*” [Larsson, 2003 as cited in (28) p. 344], much of the scholarship that was being conducted used male physiology as the norm and felt short of *truly* uplifting female athletes.

With these recent critical contributions in mind, sports science researchers have started advocating for changing the focus of scientific inquiry from physiological and biological to gendered and sociocultural constraints interacting within the performers' environment [see (22, 23, 29)]. But to reimagine sport in ways that promote gender equity from grassroots to elite levels, there is a need to better understand *why* such a shift in the research paradigm is needed.

Therefore, we turn to a brief sociohistorical overview and critical sport literature to show how and why, sport has—and continues to—uniquely restrict girls' and women's sport performance. While a comprehensive synthesis of the critical sport scholarship is outside the scope of this interdisciplinary study, a brief review integrated with existing gaps in the sport science literature highlighted in our scoping review can guide future research and practice to promote girls and women in sport (see *Call to Action*).

## Sociohistorical background

Sport as a social institution has historically been made for, and by, men (10, 11, 30). Messner (10, 11) and Nelson (30) offered sociohistorical accounts of how contemporary sport emerged in response to perceived threats to masculinity at the beginning of the 19th century. Scholars explain that organised sport became increasingly relevant within an expanding industrial capitalist order. While trends toward industrialism and capitalism created and reinforced separate, unequal spheres of life for men and women, this social order also enabled fewer men to own property and control their own labour. This political reality alongside the “perceived feminization” [(10), p. 200] taking place within public spaces prompted “a crisis of masculinity” [(10) p. 200]. In response, men [in the U.S.] organised sport and recreational opportunities in an effort to validate masculine ideals as superior [e.g., Young Men's Christian Association YMCA (31)].

Despite the use of sport to address the perceived crisis of masculinity, women challenged social exclusion and discrimination through individual and collective efforts to participate in sports (10, 13, 28, 30). In the late 19th and early 20th centuries, women claimed spaces in largely designated feminine sports such as figure skating and gymnastics (28). Women [specifically privileged white women] also gained access to golf and tennis, individual sports in which they could appear and maintain socially proper feminine displays [e.g., acting passive and composed]. Against a backdrop of broader stereotypic beliefs that viewed girls' and women's participation in sport as violating the dominant feminine ethos, persistent gender inequities eclipsed these initial gains (13, 30). Inequities included limited participation opportunities due to anxieties about physically active women appearing too masculine and medical myths that purported females as vulnerable to over-exhaustion during strenuous sports [e.g., (32)].

In the mid-and latter half of the 20th century, significant inroads would be made for women's participation in sport alongside new and persisting setbacks to gender equality (11, 13, 33, 34). Political and legal changes [e.g., the passage of Title IX in 1972 in the U.S., Sex Discrimination Act in 1975 in Britain, or the Swedish Sport Confederation's support of women's sport] directly led to dramatic increases in girls' and women's participation rates and funding within sports (11, 28). Even so, these upward trends were not ubiquitous as new barriers to gender equality emerged [e.g., the decline in women head coaches in U.S. colleges/universities (35). These, and other, structural inequities continue to reinforce women as inferior to men and sport as a man's world [e.g., women's sports must be identified such as the *Women's* World Cup (13)] even with increased opportunities and attention for elite female athletes.

These selected historical points are far from comprehensive but illustrative of how sociocultural constraints are deeply entrenched in sport and society. This sociohistorical reality further reinforces Newell's model and supports our previous argument: *sociocultural constraints are deep-rooted and impact motor development.* To achieve equity will require awareness to historical and current inequities and how they constrain female athletes from grassroot to elite levels.

### Gender as a social construction

Critical sport researchers have made significant contributions to the knowledge base by centring on issues related to gender, power, and athlete outcomes [e.g., performance or well-being (36–38)]. These scholars account for broader sociohistorical context and tend to reject views of gender as innate [i.e., attributes and actions as based on sex]. Although popular beliefs may designate behaviours either as natural due to sex [male or female] or as inherently masculine or feminine, scholars who view gender as a social concept, or construction, argue that social norms and dynamics (re)produce what attributes, actions, and activities are deemed “masculine” or “feminine” (10, 11, 36). Gender thus regards socially-agreed-upon beliefs about performance, development, and behaviour that are appropriate given an athlete's gender identity.

This supports our earlier point that sports are classified as masculine, feminine or gender-neutral [see (25, 39)] based on these historical expectations. These norms often designate girls and women athletes as physically inferior relative to boys and men. Girls and women are presumed to exhibit attitudes and behaviours that fit the feminine ideal [e.g., being less aggressive and more emotional] which are also the perceived characteristics of feminine sports [see (40)].

Prevailing gender expectations also compel women [and men] to engage in socially proper ways and gender-appropriate sports. When women athletes show stereotypically masculine qualities [e.g., competitiveness], their femininity may be questioned and they may experience marginalisation (37, 41). Such dominant views thus impact women's participation and performance—and by extension reinforce gender differences as strictly biological.

Therefore, we conducted a scoping review to synthesise existing sport science literature that adopted Newell's model to investigate expert performance. We aimed to examine the consideration given to the sociocultural constraints when assessing elite performance outside sport psychology literature. We also mapped out the extent to which relevant sport science literature provides key study characteristics and offers a gender-centred approaches to elite performance, which is key for further progressing [female] sport. Our scoping review offers a useful starting point to probe *the place that elite sport has for women in the male preserve of sport* as an attempt to promote gender equity in sport.

## Applications of Newell's (1986) model: a scoping review

We implemented a rigorous methodology to conduct the scoping review following PRISMA-ScR checklist [e.g., identifying objectives, utilising inclusion criteria, conducting searchers, processing data and synthesising results, etc. (42)] that has been developed based on the initial recommendations and frameworks for scoping reviews [see (43–45)].

### Search and study selection

Six databases were searched: SPORTDiscuss, SCOPUS, ScienceDirect, Web of Science, OVID and Google Scholar. The following search strategy was implemented:

Newell* AND constraint* AND (performance OR sport*)

There were six inclusion criteria: (1) Studies were conducted post-1986 after Newell's model was published; (2) Explicit mentioning and application of Newell's model; (3) The full text was available in English; (4) Original empirical works; (5) The sample consisted of elite or proficient performers OR there was a clear comparison between experts and novices; and (6) The study was conducted within sport utilising an expertise-requiring task.

**Figure 1:**
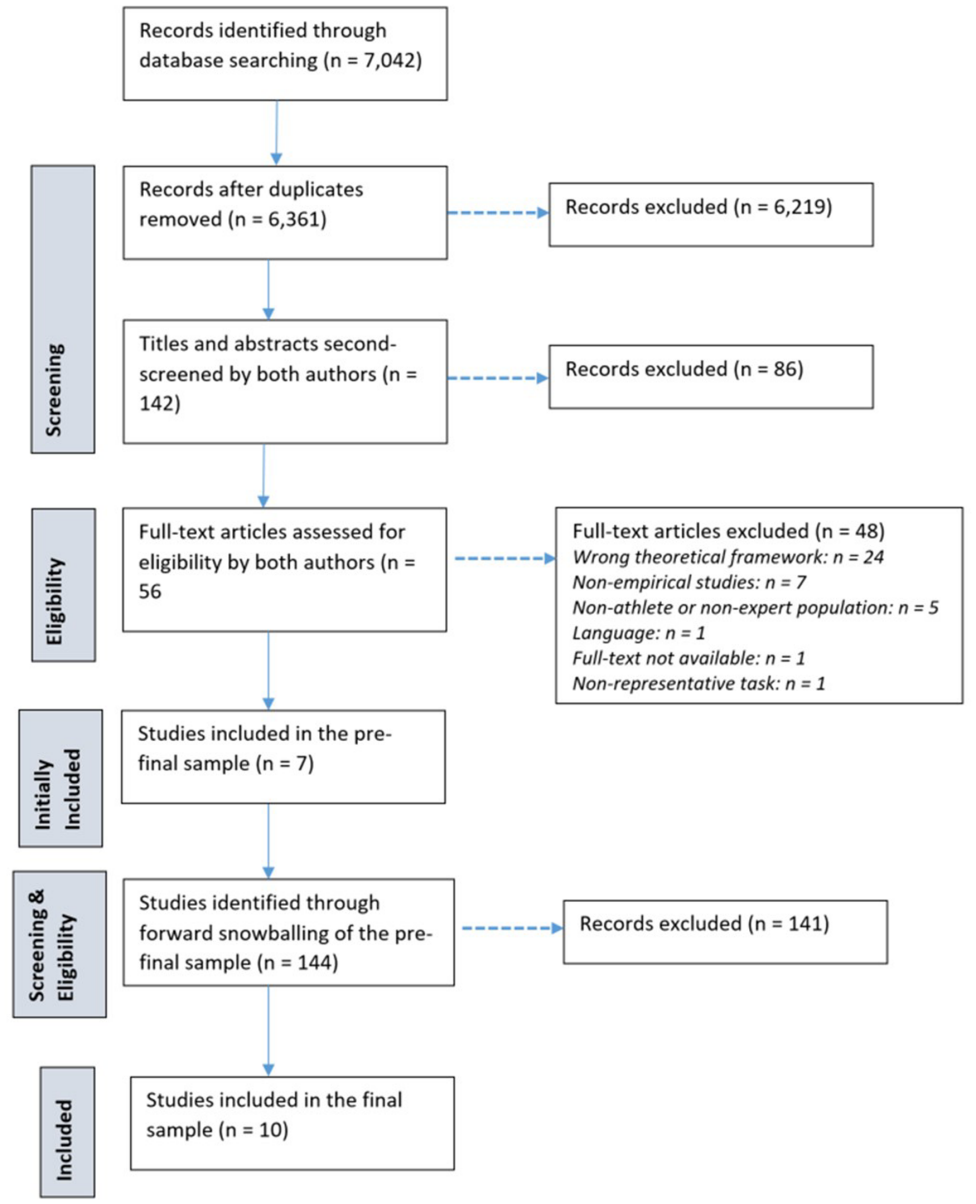
A systematic decision-making flow diagram.

The lead author identified articles, downloaded and exported them to EndNote X9 on 10th January 2020 which underwent a three-stage screening (see Figure 1): (1). Pre-screening titles and abstracts [the lead author only]; (2). Screening titles and abstracts of the pre-screened articles [both authors applying a blinded approach using Covidence; Veritas Health Innovation, Melbourne, Australia. Available at www.covidence.org]; and (3). Screening full-text [both authors applying a blinded approach using Covidence; see Figure 1]. We used the six inclusion/exclusion criteria to reach the final agreement [*ĸ* = 1]. Once the final studies were identified, we implemented the forward snowballing technique on 2nd July 2020 to identify newer publications [i.e., the *cited by* function in Google Scholar].

### Data extraction

Once the final sample was identified and agreed upon, the lead author read the identified articles in full and extracted the following study characteristics: authors, date, location, aim, study focus aligned with Newell's model, research philosophy, methodological details [i.e., sample size, sample characteristics and social identity, materials and apparatus, task, variables, and analysis], results, and discussion concerning Newell's model. The second author extracted data from one study and screened through remaining studies to compare for consistency, accuracy and level of detail as part of inter-rater reliability (45, 46). Since we included peer-reviewed studies only, we did not include a separate quality appraisal that is part of the PRISMA-ScR (42). After key data was extracted, we followed Rienhoff et al's framework (47) and further synthesised and mapped the obtained data against Newell's Model for deeper analysis.

## Results

The scoping review resulted in 7,186 studies, ten of which met the inclusion criteria and were selected for data extraction. All studies were published between 2007 and 2020 in Western countries [Australia = 4, England = 1; France = 2, Portugal = 2, Wales = 1], with six studies published between 2017 and 2020. Table 1 summarises key study characteristics and results of the identified studies.

**Table 1:** Brief summary of the included studies.

Study	Location (Lead Author)	Main Research Focus	Sample	Sample Characteristics and Social Identity	Sport and Classification (M, F, N, N/A)	Methodology	Key Results
Bideault et al, 2013 (48)	*France*	Changes in arm coordination with an increased swimming speed	*n* = 63	*Sex*: 48M & 15F *Mean age:* M = 20.9 ± 3.7 years; F = 19.4 ± 3.4 years *Role*: athletes *Expertise*: International: *n* = 13 (10M & 3F) National: *n* = 36 (26M & 10F) Inter-regional: *n* = 14 (12M & 2F) *Average Body Mass:* M = 81.2 ± 6.3 kg; F = 57.2 ± 5.4 kg *Average Heights:* M = 1.87 ± 0.04 m; F = 1.67 ± 0.06 m *Demographic data:* n/a	Swimming (*N*)	Repeated-measures; Video analysis of 7 speed bouts swum at different paces with 4 min rest intervals s	Sex differences in training history for distance speciality (endurance vs. sprint), reached speed (males reached a lower speed that results in less drag) and stroke length (males had a longer stroke). Skill-based differences as elite swimmers reached higher speed and efficiency.
Brazil et al, 2020 (49)	*Wales*	Biomechanical understanding of overload and exercise specificity on lower joint	*n* = 6	*Sex*: males *Mean age:* 23 ± 4 years *Role*: athletes *Expertise:* 100 m Personal best varied between 10:64–11:00 s. *Average height:* 1.82 ± 0.06 m *Average body mass:* 78.52 ± 6.91 kg *Average leg length:* 0.90 ± 0.03 m *Demographics:* n/a	Track and field (sprint; *N*)	Repeated-measures; 3D external force and kinematics analysis for each movement phrase performed during the 3 sessions.	While exercises were different from the block start, they promoted relevant self-organisation of coordination patterns suggesting the need to consider individual responses to task-specific coordination when selecting exercises.
Burnie et al, 2018 (50)	*England*	Explore elite coaches’ professional philosophies about to strength training and its transfer to performance	*n* = 13	*Sex*: 12 M & 1F *Mean age:* n/a *Role*: 11 coaches & 2 athletes *Expertise:* international level: 2.5–31 years of experience of coaching; 2 athletes: >12 years of experience at international level *Demographics:* n/a	track cycling (M), BMX (N/A), sprint kayaking (M), rowing (M) and athletics sprinting (*N*)	Cross-sectional semi-structural interviews	Task-specific strength was important and best achieved in a combination with non-specific strength and resistance training. The transfer is a complex process where fatigue and movement coordination were key.
Figueiredo et al, 2012 (51)	*Portugal*	Variability in arm coordination and individual profiles	*n* = 10	*Sex*: males *Average age:* 21.62 ± 2.4 years *Role*: athletes *Expertise*: competitive background: 11.9 ± 3.5 years *Average height:* 185 ± 6.8 cm *Average Body Mass:* 76.4 ± 6.1 kg *Average arm span:* 188.7 ± 8.4 cm *Demographics*: n/a	Swimming (*N*)	Cross-sectional; 3D & video analysis using anatomical landmarks during 1 max effort swim, post-swim lactate peak (repeated-measures)	Fatigue resulted in changes to coordination (e.g., speed) but coordination remained stable despite drag forces. Distance speciality explained the differences in stroke mechanics.
Figueiredo et al, 2013 (52)	*Portugal*	Relationships between efficiency parameters, energy cost and arm coordination	*n* = 10	*Sex*: males *Mean age:* 21.62 ± 2.4 years *Role:* athletes *Expertise*: competitive background: 11.9 ± 3.5 *Average height:* 185 ± 6.8 cm *Average Body Mass:* 76.4 ± 6.1 kg *Average arm span:* 188.7 ± 8.4 cm *Average % of adipose tissue:* 10.1 ± 1.8% *Demographics:* n/a	Swimming (*N*)	Repeated measures; 3D & video analysis using anatomical landmarks during 2 max effort swims 90 min apart, and another 2 swims 24 h later, VO_2_ max, blood lactate (4 measures after each trial)	Changes in coordination patterns (i.e., technique) in response to fatigue (e.g., decreased speed, etc.) as a means to optimise efficiency given reduced power output suggesting that adaptations to technique are influenced by several constraints.
Oppici et al, 2017 (53)	*Australia*	Effects of deliberate practice under different constraints on perceptual skills and visual attention	*n* = 48 (initial) *n* = 37 (final)	*Sex*: males *Mean age:* <13 years (9 futsal and 8 football players), <15 years (8 futsal and 11 football players) *Expertise*: 6.8 ± 1.2 years of experience in football (*n* = 20) and 7.0 ± 1.6 years of experience in futsal (*n* = 17). *Training History*: approx. 400 competitive games per group; approx. 1,220 h and approx. 1,260 h of domain-specific structured practice per group respectively *Demographics*: 1 Australian and 1 Spanish Group	Futsal (N/A) and football (M)	Cross-sectional; Video analysis and eye-tracking during 6 modified games with 5 min rest (each player was tracked during 1 game only after taking a rest).	Futsal players primarily looked at other players for longer, maintained visual attention on other players and were switching attention between the players and the ball more frequently. Soccer players were scanning the field more frequently while futsal players played at higher intensity suggesting that task constraints influence the development of skills.
Piggott et al, 2020 (54)	*Australia*	Examine if an interdisciplinary approach offers a more comprehensive understanding and better predictions of individual match performance	*n* = 59 (initial) *n* = 21 (final)	*Sex:* males *Mean age:* 21.27 ± 3.11 years *Expertise*: Semi-professional league club; senior playing squad *Training History:* Average matches played per season: 15.6 per player *Average Height:* 186.79 ± 7.17 cm *Average Body Mass:* 84.0 ± 9.13 kg *Demographics:* n/a	Australian Football (M)	Cross-sectional mixed methods; Physiological assessments using sprint repeats, coaches’ evaluation of mental toughness after each small-sided game	All three disciplines were significant predictors of a match disposal efficiency; performance in small-sided games predicted coach scores and was explained by group performance. Interaction of constraints as better coping skills with psychological pressure allowed making more accurate decisions on how and when to apply physical capabilities.
**Seifert et al, 2007 (55)	*France*	The effects of organismic, environmental, and tasks constraints on arm coordination adaptations	*n* = 42	*Sex*: 30M & 12F *Gender*: cis-gender *Average age:* n/a *Expertise*: national (M) and international level (M & F) *Skill level*: Average % of a Word Record on 100 m freestyle per group (elite men = 93.6 ± 1.7%; mid-level men = 86.1 ± 2.1%; elite women = 91.7 ± 4.2% *Average Body Mass*: elite men = 76.5 ± 5 kg; mid-Level men = 74.3 ± 3.9 kg; elite women = 58 ± 4.9 kg A*verage Heights:* elite men = 184.4 ± 4.9 cm; mid-level men = 183.2 ± 3.5 cm; elite women = 169.6 ± 5.1 cm *Average Arm Span:* Elite men = 192.8 +/− 5.2 cm; mid-level men = 189.4 ± 5.4 cm; elite women = 175.2 ± 4.6 cm *Demographics:* n/a	Swimming (*N*)	Repeated-measures; Video analysis of 7 self-paced swims at different velocities	Sex differences explained higher stroke frequency and length in elite males vs. females while differences in expertise explained these differences between elite and mid-level males. Expertise explained changes in stroke length with changed distance. However, individual-structural differences should be considered due to the indirect effect of creating drag and resulting in individual adaptations to coordination (technique).
*Woods et al, 2019 (56)	*Australia*	Examine and compare some constraints affecting ball disposal for later interdisciplinary evidence-based	*n* = 45	*Sex*: males *Expertise*: elite (AFL) and semi-elite (AF) *Competitive Experience*: At least one season in the respected league *Demographics:* n/a	Australian Football (M)	Longitudinal cross-sectional observational design Video analysis of 40 games (22 AFL and 18 AF games).	Expertise-based differences in possession time as elites were quicker and more dynamic to dispose the ball, and held on to it for longer and under more pressure suggesting more prominent individual-functional constraints as task constraints/demands increase.
Woods et al, 2019 (57)	*Australia*	Identify key constraints in training	*n* = 3	*Sex*: males *Expertise*: >5 years of coaching experience *Demographic data:* n/a	Australian Football League (M)	Mixed methods Semi-structured interviews and grounded theory to develop a constraints-led framework for kicking performance that was applied to video analysis of 10 AFL matches	Individual and environmental constraints were the most and least representative in predicting kicking performance respectively (out of 12 constrain comparisons) implying the need for greater contextualisation during training.

* Participants’ sex was not reported.

** Participants’ gender was assumed based on the language used; yrs, years; M, males or masculine; F, female or feminine; N, gender-neutral sport; N/A, sport classification is not available (25, 39).

### Research focus and study design

Overall, no studies centred around a female athlete, the unique demands the female athletes experience, approaches to female athletes or perspectives of female coaching/support staff. Only Oppici et al's (53) primary focus was on psychological aspects of skill acquisition and motor control (i.e., perceptual-cognitive and perceptual-motor skills) that were examined using a quantitative repeated-measures design with male athletes.

The single qualitative study in our sample (50) investigated the coaching philosophies using cross-sectional semi-structured interviews following constructivist epistemology and post-positivist research paradigm. No other studies reported the underpinning research philosophy and the guiding paradigm.

Nine studies focused on biomechanical or physiological research questions and implemented a combination of quantitative measures techniques using a repeated-measures design (48, 49, 52, 55, 56), a between-participants cross-sectional design (54) or a mixed-methods cross-sectional design (57) (see Table 1).

#### Sample characteristics and social identities

Overall, 202 participants (126 males, 28 females) across the ten studies were included in the final analysis. Five studies (49, 51–54) investigated males only, and three studies (48, 50, 55) used a mixed-sex sample. The average age ranged from 13.6 to 21.62 years for male participants and from 19.4 to 19.6 for female participants. Neither Burnie et al. (50) nor Woods et al. (56, 57) provided the average age of their participants. Two studies (50, 57) reported coaches' perspectives while eight studies reported athletes' perspectives.

Six studies (48, 49, 51, 52, 54, 55) reported participants' anthropometric measurements some of which were relevant to research questions [e.g., average body mass or arm span]. These studies used anthropometric measures to describe the sample (54), estimate dependent variables (49) or explain the reported performance difference among sex (55).

None of the identified studies reported participants' social identities [e.g., gender identity, ethnic identity, religious beliefs, disability status] nor sociodemographic characteristics [e.g., socioeconomic status, marital status, level of education, etc.]. Only Oppici et al. (53) reported nationality associated with the location [i.e., Spanish and Australian groups], while Seifert et al. (55) assumed cis-gender identity based on the single-sex categories the participants competed in [i.e., female swimmers were classified as elite women]. Woods et al. (56, 57) reported neither the gender nor sex of their participants. Since the authors investigated AFL players and coaches, it is assumed all participants were male given the overall AFL history in Australia (58).

#### Context

Based on the existing gendered sport classification (25, 39), five studies investigated gender-neutral sports [i.e., swimming (51, 52, 55) and track and field (48, 49)], four studies investigated masculine sports [i.e., futsal and European football (53) and Australian Football (54, 56, 57)] and Burnie et al. (50) investigated a mixture of gender-neutral and masculine sports such as sprint kayaking, BMX cycling and athletics sprinting. No studies investigated feminine sports. Nor did a single study investigate female athletes in masculine sports such as European football or rugby.

### Mapping against the Newell's model of constraints

**Figure 2:**
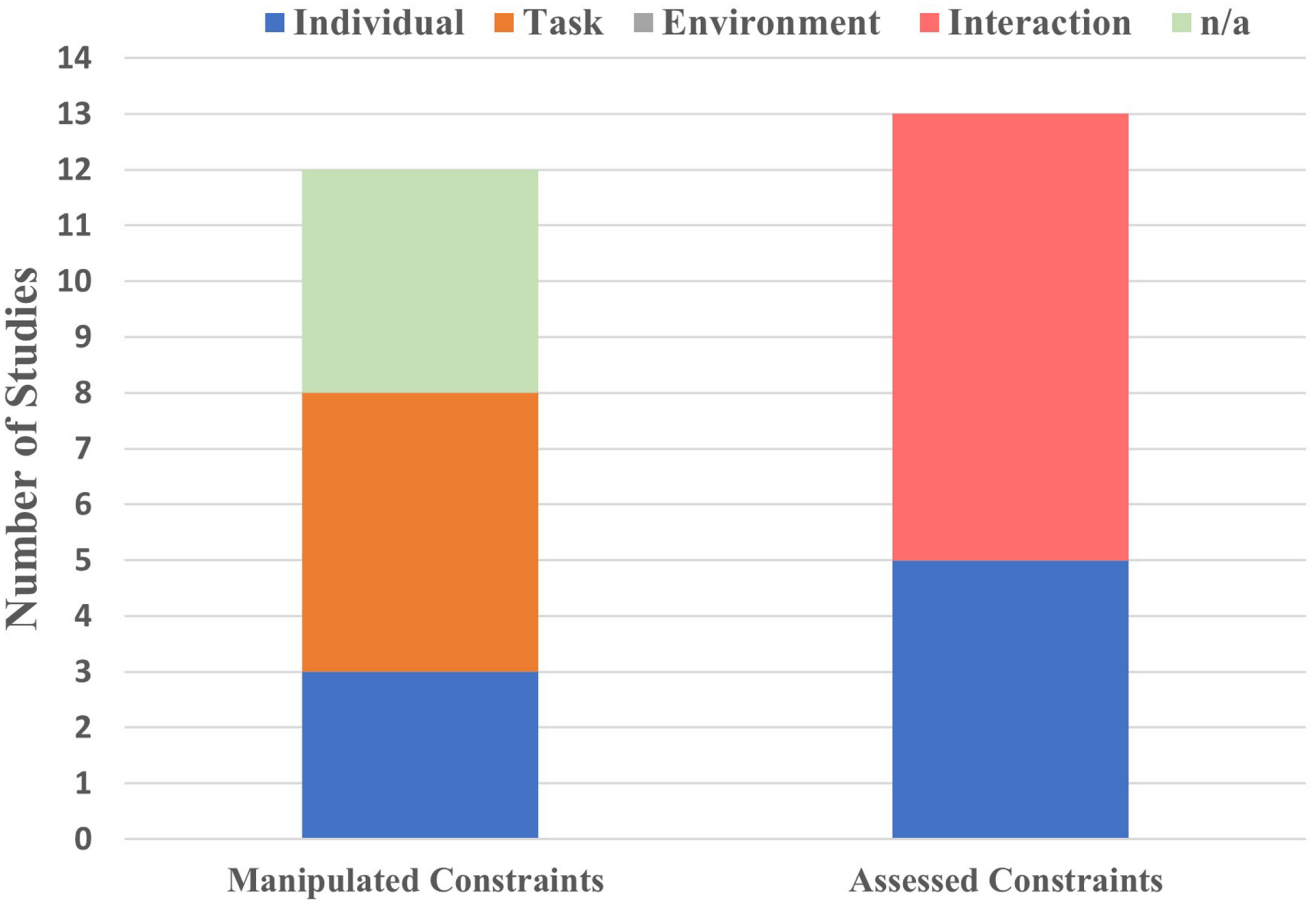
A summary of study designs mapped against the Newell's model of constraints.

**Figure 3:**
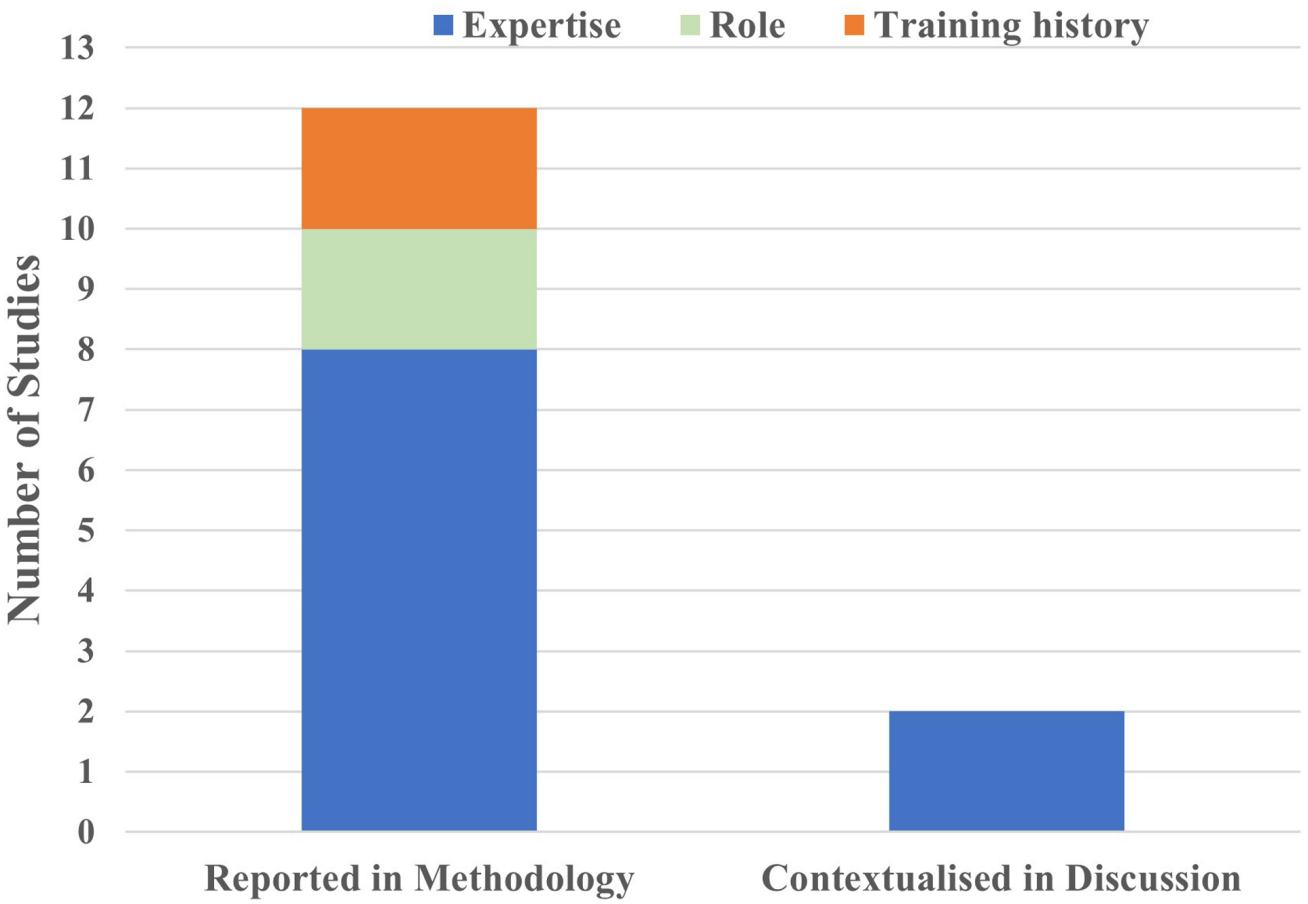
Summary of sociocultural constraints.

Scoping review synthesises and maps key concepts and evidence in relation to the research question (59). Building on PRISMA-ScR guidelines (42), we further synthesised the extracted data against Newell's model following the adapted approach from Rienhoff et al's (47) systematic review [e.g., the explicit mapping against the sociocultural constraints]. All studies were re-categorised regarding what constraint was manipulated and assessed in relation to the study design. We also looked at sociocultural constraints and the extent to which these were considered in each study (see Figures 2, 3).

#### Manipulated constraints

Five studies (48, 49, 51, 54, 55) manipulated task constraints such as a pitch size or swimming pace to assess their effects on the interaction of constraints [i.e., technique or movement coordination]. Two studies (51, 52) manipulated individual-functional constraint (i.e., fatigue), and one (49) manipulated individual-structural constraint [i.e., strength]. Four studies (50, 53, 56, 57) made no manipulations in line with their research focus (see Figure 2).

#### Assessed constraints

Eight studies (48, 51–57) assessed the interaction of constraints [e.g., passing skills, performance or stroke characteristics] as a result of experimental manipulations, five (52–56) out of which also assessed task or individual constraints [e.g., perceptual skills, sex differences or physical abilities]. Only Seifert et al. (55) investigated sex differences and noted that sex was a confounding variable in relation to the physiological differences, which, in turn, affect the environmental factors [e.g., bigger body size and differences in body fat distribution in male swimmers create different water responses or drag compared with female swimmers].

#### Sociocultural constraints

All studies reported participants' skill level or expertise and training history as part of the sample characteristics. Despite this, only Seifert et al. (55) and Woods, Jarvis and McKeown (56) contextualised expertise-based differences, as both authors argued that expertise influenced other constraints that led to better performance [or a more efficient coordination pattern]. Nonetheless, these studies (55, 56) failed to make links between sample characteristics and participants' social identities and further discuss how sociocultural constraints might have impacted training opportunities and therefore, training history and the development of skills. Instead, the level of expertise [e.g., professional, semi-professional, elite, mid-level, etc.] was used as an independent variable to categorise participants and make group comparisons [e.g., (54, 55, 57)].

Similarly, Seifert et al. (55) failed to examine sex differences from a gendered perspective to further contextualise the reported performance similarities in swimming. In essence, sociocultural constraints were decontextualised as no consideration for the potential sociocultural reasons beyond group comparisons was offered across the identified studies (see Figure 3).

## Discussion

We aimed to synthesise and rigorously examine the extant sport science literature on applying Newell's model when examining elite performance and whether it has considered the effects of sociocultural constraints. Our results highlighted the limited application of the constraints-led approach in elite performance [i.e., 10 studies were included in the final sample], none of which examined the intertwined nature between sociocultural constraints and the obtained results. We adopted an interdisciplinary approach to interpret synthesised results considering a sociohistorical perspective and critical sport research so that researchers and practitioners can consider *how* to minimise the gender gap in elite sport based on female evidence.

Our results revealed the peripheral treatment of psychological over physiological and task-specific variables, despite Balagué et al's (60) point that *all* constraints are relational to each other and cannot be mutually exclusive. This finding is in line with Johnston et al. (61) results which identified over-reliance on physiological profiles across sport science literature.

Similar to Walton et al's (62) systematic literature review of sport psychology literature, our findings also revealed a gender imbalance across sport science literature that heavily focused on male participants. This supports the ongoing argument that women's game is based on men's evidence.

Further, our results exposed sport science researchers' failure to report and/or contextualise demographic data regarding sociocultural constraints. Considering the location of the identified studies [i.e., Western countries], examined sports [i.e., masculine or gender-neutral sports] and existing assumptions of a default option in sport, we can *assume* the identified studies investigated athletes representing the presumed status quo—white heteronormative cisgender able-bodied. These results are in line with Ram et al. (63) findings, who synthesised sport and exercise psychology literature published between 1987 and 2000 and concluded the oversight of participants' sociodemographic data. While Ram et al. (63) reported a slight increase in the reports of participants' race/ethnicity and sexual orientation in comparison to Duda and Allison's (64) findings, no systematic attempts to consider participants' social identities and their effects on the development of expertise [e.g., training history] were identified in this study.

Combined with existing evidence showing that stereotypes about marginalised groups can negatively affect elite performers (65), the existing gender gap at elite levels can be explained in light of the intertwined nature of the three constraints. The overwhelming misrepresentation of psychological variables, under-treatment of sociocultural constraints and ongoing peripheral treatment of a female athlete across sport science literature [see (1, 66)] fail to advance existing knowledge and address the existing gender inequity. For example, Emmonds et al. (1) reported mere adoption of male protocols that dismiss a female athlete's physiological and biomechanical profiles and female sport culture [i.e., female sport based on male evidence]. Later Taylor, O'Connor and Hanlon (3) showed that female athletes report poorer access to expert coaches or lack of playing opportunities at a young age, while players from lower socioeconomic backgrounds reported being denied access to the facilities that influence physical and cognitive development resulting in the developed expertise.

As such, our results further support Cummins et al's (29), Fox et al's (22) and later Parsons, Coen and Bakker's (23) critique that such an oversight fails to provide a full picture of one's (under)performance and calls for changing the focus of scientific inquiry. These points also highlight the default and preferred option among sport sciences to be a white cisgender able-bodied heterosexual male playing mainstream masculine sports. There *still* is a need to address the misrepresentation of minoritized populations across a broader variety of sports despite the ongoing calls to do so [see (63, 64, 67)] and critical sport research revealing contextual and cultural factors that impact performance.

For instance, researchers have adopted various critical [i.e., non-dominant] theoretical approaches [e.g., poststructuralism, intersectional feminism, and cultural praxis] to centre women's experiences in male-dominated sport settings (68–70). This research has shed light on the myriad ways in which girls and women experience and navigate marginalisation, including, but not limited to, gender-restrictive assumptions (24, 36) or stereotypical representations (71). Critical sport researchers have also attended to women's experiences at the intersection of their other social identities such as sexuality (72) and race (70, 71). This intersectional perspective has made visible the ways in which women athletes may be uniquely marginalised by interlocking oppressions [e.g., racism and sexism] given their other minoritized identities. Though more research is needed, these efforts serve as examples of how cross-disciplinary [e.g., psychosocial and motor] research in sport can, and ought to, focus on oft-ignored topics of identity, power, and gender (38) to better understand physical performance. Nonetheless, prevalent approaches to sport science fail to acknowledge these realities and chooses to focus on individual structural (i.e., physiological or sex) differences taken at face value as identified in our scoping review. Such a trend dismisses sociocultural constraints as *exploratory* variables to these differences and fails to consider a more holistic, interdisciplinary view to elite performance (73).

Extant critical sport scholarship and the research gaps that our scoping review made evident further illustrate and expand our understanding of the existing constraints asymmetry (74). Even though the physiological and biological differences between male and female athletes explain differences in performance outputs and achievements [e.g., maximum aerobic capacity, longer cycles or higher speed achieved and maintained during the performance (75)], such differences are *still* used to explain the gender gap in sport. In more recent years, Kavoura and Kokkonen (76) concluded in their scoping review that research with gender and sexual minority athletes and coaches was still lacking. Our results also support this point.

The highlighted gender and sport misrepresentation among the scholarly works and decontextualised approach to female athletes contribute towards widening the gap and (re)producing the myth of meritocracy in sport [i.e., the belief that individuals have similar opportunities to achieve success and can thus succeed through hard work (13, 28)]. This point aligns with Capranica et al's (77) invitation to address cultural and socio-political factors first before scholars can advance a rigorous scientific discussion on physiological development and differences related to performance.

The scientific oversight of sociocultural constraints and decontextualised and universalised approach in the sport science literature risk discounting possible effects of an elite athlete's class, ethnic and racial identity, gender identity and sexuality on performance (78–80). When researchers ignore these sociocultural factors, they can (re)produce the perception that sport is meritocratic and inadequately dismiss the distinct experiences that women have in sport, including their effect on performance and female athletes' developmental progression to elite sport (81).

### Limitations

As a preliminary example of interdisciplinary research informed by Newell's model for gender equity promotion, this study has two main limitations. Similar to most scoping reviews published across different sport science areas, our study was limited to scientific literature published in English and did not consider non-English and often non-Western research. A more comprehensive review that included non-Western research might have allowed for more nuanced, intersectional insights that could better account for the varied experiences of elite female athletes navigating sociocultural constraints.

Second, while Newell's model offers a clear and simple framework to assess gendered discourse and its effects on motor performance, we excluded other frameworks that are likely to address sociocultural constraints within the sport psychology literature that were outside the scope of this study (e.g., Sport, Policy Factors Leading to International sporting Success [SPLiSS] (82)]. In doing so, we highlighted how the sport science literature has fallen short of adopting a robust intersectional and interdisciplinary perspective to provide holistic athlete-centred evidence by drawing on knowledge developed across the disciplines. These points are in line with the gender gap noted by Peters et al. (81) when scoping the literature on developmental pathways to expertise among girls and women.

Sociocultural constraints should regain focus in the performer-environment-task relationship to allow sport scientists and practitioners to more adequately develop and apply female evidence to female sport. As such, we finish with a call for action to conduct research and implement practice *for* a female so that we could together identify the role sport can play *for* women to debunk the myth of meritocracy and its possible harmful implications.

## Call to action

More clearly elucidating the significance of gendered dynamics as a sociocultural constraint requires that we adopt a more interdisciplinary approach in research and consider taking more ambitious, practical *actions* targeting the longstanding problem of gender inequity in elite sport. We implore that sport stakeholders attend to the unique needs of female athletes and—more boldly—reimagine (elite) sport moving away from centring on immediate individual structural constraints and a deficit-based approach. Instead, the focus should shift towards addressing the embedded sociocultural constraints and the amplified differences through the empowerment of a female athlete. Thus, this *call to action* is aimed to guide sports science researchers, practitioners, and decision-makers to reimage sport, promote gender equity and better understand the place that elite sport can have *for* women.

### Recommendations for sport performance researchers

We contend that researchers need to engage in empirical efforts that are not simply *on,* but *for,* female athletes for the promotion of gender equity in sport (11, 83). Though science is presumed to be objective and neutral—as sport is presumed to be meritocratic —these social institutions have been historically created for and by (white, cisgender, heterosexual) men (11, 83, 84). This call to action echoes the seminal work of feminist cultural studies scholars who, almost thirty years ago, implored sports researchers to adopt a more robust women-centred agenda (34, 85). While impactful contributions within sport sociology and cultural sport psychology have been made since this early call, other sport science disciplines (as illustrated through our scoping review) have yet to make meaningful progress. We implore researchers to acknowledge gendered sociohistorical underpinnings, make contributions to the scientific literature that challenges the supposed neutrality of science and conduct sport research that contests a masculine status quo. Sport science researchers should centre the shared, unique, and intersectional experiences of female athletes from a strength-based perspective (34, 85). We propose two methodological solutions to consider.

First, researchers across all disciplines should not only conduct studies with athletic female populations but also collect and interpret demographic data of their research samples. This includes data on the protective characteristics [e.g., gender identity, religion, sexual orientation, or ethnic identity] as well as training history, exposure to professional staff and availability to access training facilities. This data should then be used to interpret their findings and consider intersectionality and sociocultural barriers to elite performance as the exploratory variables in their studies (22, 23, 64, 79, 80). Thus, sport science researchers must adopt a strength-based perspective of potential sex-based differences: viewing the unique needs of female athletes not as deficits in comparison to masculine standards, but on their own terms that, when fully supported, enable thriving (86). Such an approach is necessary in order to celebrate women's diverse yet unique strengths and at the same time seriously question male-dominated and male-defined sport (34).

Sport science researchers can specifically adopt a mixed-methods approach to investigate links between sociocultural constraints and skilled sport performance *in conjunction with* empirical and critical frameworks [e.g., poststructuralism, cultural praxis] that afford an understanding of gender as a social construction.

One possible line of inquiry could be to quantitatively assess links between opportunities and access to resources available to female athletes and actual performance outcomes; or between their perceptions of pressure due to tokenisation or perceived inferiority and objective performance at critical points. Such an understanding, to our knowledge, is currently missing, let alone considered, as highlighted in our scoping review.

This quantitative arm of research could be complemented with more information-rich qualitative data that captures female athletes' strategies and existing social supports to develop more rigorous female athlete-specific knowledge. This would help us better elucidate the varied and nuanced ways that elite female athletes negotiate gendered constraints [and supportive roles that male allies might play] to both disrupt and reproduce the masculine status quo. These research efforts need to prioritise the unique and intersectional experiences of female athletes [e.g., ethnicity or disability (34, 87)] to help surface how female athletes exercise agency in various performance contexts. Consequently, these research findings have the potential to better guide interpersonal and institutional changes in the service of gender equity promotion.

Consequently, calling for sport science that supports individual athletes in this way can create a power-filled, strength-based discourse that contests rather than upholds sexism or other forms of discrimination to empower (female) athletes to reach their full potential. Essentially, it helps us develop and adapt female evidence relevant to female sport.

### Recommendations for sport performance practitioners and decision-makers

We also call on sport performance practitioners [e.g., consultants and coaches] and decision-makers [e.g., organisational leaders] to make a more robust commitment to advocating for female athletes and promoting gender equity through sport. We offer guiding suggestions they may consider in relation to individual [i.e., personal beliefs and interactions with other stakeholders], and institutional [i.e., organisational structures and resources] aspects.

#### Individual

Practitioners and decision-makers need to critically examine beliefs about whether performance and behavioural differences are strictly inherent, and more fully attend to the long-standing sociocultural limitations that have restricted the capacity for women to close the developmental and performance gap. Sport practitioners, educators, and decision-makers can be more intentional about framing sport performance along a continuum of competence rather than on the presumed male-female binary especially attending to instances in which female athletes outperform male athletes [e.g., at early ages or skill-based sports (15, 24)]. Sport leaders are not immune to internalising beliefs that designate males as superior to females, and no change can occur until leaders challenge their own beliefs.

Therefore, we invite sport leaders to reflect on their own identities, biases, and perspectives, and how these aspects influence their practice. For instance, “why not” and flipped questions are practical strategies that sport professionals can use to better recognise their biased thinking and challenge status quo masculine beliefs (88). A sport professional might ask themselves, “If she was a man, would I have responded differently?”, or “If she was a white woman, would I have thought the tone was bossy?”. Indeed, Rees and Salvatore (89) showed that simple questioning of those stereotypes was an effective strategy in disrupting the stereotype threat and maintaining performance in sport.

Sport leaders also need to be critically attentive to the sociocultural constraints that female athletes must negotiate in male-dominated performance domains. When an athlete endures social discrimination, their health, experience, and performance will be negatively affected (22, 38). Awareness of these factors is vital for professionals to understand the challenges faced by (their) female athletes. The burden of educating or making others more aware of their marginalisation should not fall on female athletes. Instead, male sport leaders need to take initiative to develop their own critical awareness of the overt and covert ways women experience marginalisation in sport. Male sport practitioners and decision-makers can educate themselves using peer-reviewed and lay media resources [e.g., *Just Women's Sports* media company] that uplift female athletes' and stakeholders' experiences. Through these trusted relationships, practitioners can *ask* rather than *assume* they know about each female's experience and avoid overgeneralisations that negatively affect athletic performance (65, 84). These interactions should be framed as an invitation for female athletes to share about their experiences on their terms rather than feeling burdened with the responsibility to educate. Male practitioners can listen, *try* to understand and ask about ways they can support female athletes and stakeholders in their organisations [e.g., “what does support look like for you?”]. Developing an awareness of the myriad barriers that female athletes experience and the ways they want to be supported is a critical step toward addressing unconscious biases, and preconceptions, and acting to advocate for gender equity.

With a sharper critical sex-and-gender-centred lens, male sport leaders can more fully acknowledge that sexism uniquely limits female athletes instead of accepting them as an inherent part of the competition and then act to challenge instead of compelling female athletes to deal with sexism. Male professionals have a unique responsibility to use their social standing and structural advantages to interrupt derogatory behaviours and practices in their interactions to advantage *all* participants. As decision-makers, they need to advocate for increased representation of women in leadership positions to create a more balanced representation. If sport performance professionals truly aim to optimise their athletes' performance, they must create environments that offer more balanced perspectives (in both male and female sports settings) through diverse representation. Incorporating females into coaching and leadership positions to work with male and female athletes can better ensure that athletes are challenged through the specific demands of their sport instead of calling for females to “do better” and adapt to sexism.

#### Institutional

Sport organisations, clubs, and associations must also push for institutional changes that support reimagining the male-dominated landscape. A long legacy of sexism has disadvantaged female athletes—limiting their access to participation, quality opportunities, and resources that enable high performance. Continued efforts for equitable investment in female athletics must include organisational and institutional initiatives that create and grow sustainable grassroots opportunities for girls and young female athletes to develop skills vital to compete at more elite levels. Alongside efforts to improve the quality and quantity of pathways for female athletes to compete at elite levels, organisations can create developmental opportunities for female athletes to transition from athletes to all other professional roles including coaches, referees, performance directors, practitioners, and team owners. Various developmental pipelines can allow women to further cultivate their passion for sport and occupy positions of power within professional organisations to ensure that sufficient resources are allocated toward gender equity promotion at *all* levels. However, developmental pipelines need to have a clear established pathway toward the decision-making and leadership positions to influence change.

These initiatives to get more women in (male-defined and centred) sport alone, however, are not enough because quality performance defined by masculine standards should not be the ultimate goal (34). Organisational leaders must challenge industry practices that treat female-designated sports as less worthy of investment—including material resources [e.g., sport facilities or equipment], financial sponsorship or media coverage. Interscholastic programmes, associations, and sport organisations and clubs can create and adequately fund opportunities for female [and male] participation in sport beyond those deemed gender appropriate to broaden and enhance individual athletes’ capacities and our collective sport potential. Such initiatives, in turn, will help to challenge the existing stereotypes which often inhibit physical performance and overall sport experience.

Ensuring that sport is an empowering context for all athletes to optimise their capacities requires that sport leaders acknowledge how female athletes have been and continue to be constrained within male-dominated sport. Wholescale change requires a coalitional effort to contest sexist culture. Sport leaders must make a more robust commitment to gender equity in and through sport. And so does sport science research.

## Data Availability

The original contributions presented in the study are included in the article, further inquiries can be directed to the corresponding author/s.
